# Investigation of alkaline hydrogen peroxide pretreatment and Tween 80 to enhance enzymatic hydrolysis of sugarcane bagasse

**DOI:** 10.1186/s13068-019-1454-3

**Published:** 2019-05-03

**Authors:** Hongdan Zhang, Shihang Huang, Weiqi Wei, Jiajie Zhang, Jun Xie

**Affiliations:** 1 College of Forestry and Landscape Architecture, Key Laboratory of Energy Plants Resource and Utilization, Ministry of Agriculture, South China Agricultural University, Guangzhou, 510642 People’s Republic of China; 2 CAS Key Laboratory of Renewable Energy, Guangzhou Institute of Energy Conversion, Guangzhou, 510640 People’s Republic of China; 3 College of Light Industry and Food Engineering, Nanjing Forestry University, Nanjing, 210037 People’s Republic of China

**Keywords:** Sugarcane bagasse, Alkaline hydrogen peroxide pretreatment, Glucose, Tween 80

## Abstract

**Background:**

Due to the intact structure of lignocellulosic biomass, pretreatment was a prerequisite to improve the enzymatic hydrolysis by disrupting the recalcitrant lignocellulose and increasing the accessibility of cellulose to enzyme. In this study, an alkaline hydrogen peroxide (AHP) pretreatment of sugarcane bagasse with various loadings of H_2_O_2_ (1.25–6.25 wt%) at temperatures of 60–160 °C was proposed to degrade hemicellulose/lignin and improve the enzymatic digestibility.

**Results:**

It was found that increasing H_2_O_2_ loadings during pretreatment lead to the enhancement of substrate digestibility, whereas the alkali (only NaOH)-pretreated solid generated higher glucose yield than that pretreated under AHP pretreatment with lower loading of H_2_O_2_. This enhancement of enzymatic digestibility was due to the degradation of hemicellulose and lignin. Furthermore, Tween 80 was added to promote enzymatic digestibility, however, the increased yields were different with various substrates and hydrolysis time. The highest glucose yield of 77.6% was obtained after pretreatment at 160 °C for 60 min with 6.25% H_2_O_2_ and the addition of Tween 80, representing 89.1% of glucose in pretreated substrate.

**Conclusions:**

This study demonstrated that the AHP pretreatment could greatly enhance the enzymatic saccharification. The addition of Tween 80 played remarkable performances in promoting the glucose yield during enzymatic hydrolysis by stabilizing and protecting the enzyme activity. This study provided an economical feasible and gradual process for the generation of glucose, which will be subsequently converted to bioethanol and bio-chemicals.

**Electronic supplementary material:**

The online version of this article (10.1186/s13068-019-1454-3) contains supplementary material, which is available to authorized users.

## Background

Declining fossil fuels, higher demand for energy, and serious environmental problems had led to attempts to identify sustainable fuels derived from lignocellulosic biomass. Sugarcane bagasse is the fibrous material obtained after juice extraction from sugarcane [1]. Generally, they were discarded away, or fed into mill boiler, which were not favorable for the sustainable development of resource and environment [2]. The abundant carbohydrates (such as cellulose and hemicellulose) and lignin in sugarcane bagasse are promising sources for the production of value-added chemicals and fuels by bio-refinery processes. However, the rigid and intact structure of lignocellulosic biomass made it difficult to break down to hexose and subsequently ferment to bio-based fuels and chemicals. Consequently, several steps including pretreatment were proposed to enhance the enzymatic digestibility by breaking the structure down and improving the accessibility of enzyme to cellulose [3, 4].

Recently, various pretreatments using dilute acid/alkali, liquid hot water, ethanol, and ionic liquids had been proposed [5]. Numerous research have concentrated in alkaline pretreatment due to the lower work temperature and pressure, less corrosion, simpler reactors, the reuse of residual alkali, minimal formation of inhibitors, and preserving carbohydrates considerably [6, 7]. Alkali pretreatments could enhance the enzymatic digestibility by swelling fibers, breaking ester bonds of lignin-carbohydrate complexes, solubilizing lignin molecules, increasing the surface area, and providing more cellulose for the accessibility of enzyme. Among the alkali pretreatments reported so far, AHP pretreatment was appealing to the effective degradation of lignin from lignocellulosic biomass because H_2_O_2_ could degrade to oxygen and water without any residues left in the process [8]. Li et al. [6] investigated the plant cell wall recalcitrance features of diverse bioenergy feedstocks during AHP pretreatment with 12.5–50 wt% H_2_O_2_, suggesting that major alterations in structure, compositional organization, and enzymatic hydrolysis in goldenrod and corn stover rather than in hybrid poplar. The ultrasonic-assisted alkali peroxide pretreatment of Jerusalem artichoke stem released 79.4% of hextose and showed a highest total sugar concentration of 10.4 g/L, which were 2.4 and 2.6 times higher than that obtained from the control, respectively. This enhancement was ascribed to the distinctive extraction of pectin polymers and lignin, accompanying with great alterations of cellulose degree polymerization (DP) and crystalline index (CrI) [9].

To achieve high cellulose digestibility, AHP pretreatment were carried out with relatively high reaction temperatures, large amount of chemicals, or sequential two-stage pretreatments. It was reported that AHP treatment of *Douglas fir* at 180 °C for 30 min with 10 wt% loading of H_2_O_2_ generated cellulose-to-glucose yield of 95% [10]. The alkaline peroxide delignification of corn stover were conducted at 50 °C for 3 h over the range of 3–50 wt% H_2_O_2_, when the H_2_O_2_ loading reached 25 wt%, about 90% of glucose and 80% of xylose were liberated after a digestion period of 120 h [11]. Yuan et al. [12] investigated the bamboo pretreatment with NaOH pre-extraction (8 wt%) at 100 °C for 3 h followed by AHP with 4 wt% H_2_O_2_ at 75 °C for 3 h, suggesting that the pretreated substrate recovered 87% of the inherent sugars in native bamboo with enzyme loading of 9 FPU/g cellulose. Though visible strides had been made in evaluating the influence of AHP pretreatment on the deconstruction and enzymatic digestibility of lignocellulosic biomass, a comprehensive picture of AHP pretreatment under large-scale temperatures with different dosage of H_2_O_2_, and the comparison with only NaOH pretreatment on the enzymatic hydrolysis and how they affected the cellulose accessibility was still unclear.

It is generally recognized that the addition of additives such as non-ionic surfactants, polymers and non-catalytic proteins could enhance the enzymatic hydrolysis, reduce the enzyme loading, or shorten the hydrolysis time [13–15]. Yan et al. [14] reported that by adding BSA to dilute acid pretreated poplar generated more than 90% glucose and 95% xylose during enzymatic digestibility process. In our previous study, the addition of Tween 80, BSA, PEG 4000, sulfonated lignin, and organosolv lignin on enzymatic hydrolysis of pretreated substrates were compared, indicating that Tween 80 performed excellent increment in liberating glucose [13]. The boost to enzymatic digestibility by Tween 80 was due to its capacity in swelling fibers, increasing surface area, and improving the cellulase accessibility to cellulose [15].

Though the positive influence of Tween 80 on the enzymatic saccharification had been reported in previous research, the comparison saccharification of NaOH and AHP-pretreated substrates with the addition of Tween 80 under different temperatures were scarce. Thus, the objective of this study was to investigate the influence of AHP pretreatment under various conditions (60–160 °C, 1–4 h, 0–6.25 wt% H_2_O_2_) on the saccharification of pretreated substrates. Furthermore, the structural modifications were evaluated to determine their influence on saccharification using X-ray diffraction (XRD), scanning electron microscopy (SEM), fourier transform infrared spectroscopy (FT-IR), and thermogravimetric (TG) analysis. In addition, the effect of Tween 80 on the enzymatic saccharification of AHP and NaOH-pretreated substrates were investigated to determine their mechanism.

## Results and discussion

### Compositional analysis after pretreatment

The composition of sugarcane bagasse pretreated using NaOH and AHP pretreatment with various temperatures and their influence on solid recovery, cellulose retention, and the degradation of hemicellulose/lignin were summarized in Table 1. As shown, when the sugarcane bagasse was pretreated with only 3% NaOH at 60 °C for 4 h, the solid recovery was 90.4%, the removal of xylan (25.5%) and AIL (11.9%) contributed to the solid loss, resulting in the reduced contents of xylan and AIL, and the increment of glucan content in pretreated substrate. Meanwhile, the glucan recovery reached 98.5%. As the pretreatment temperature was elevated from 60 to 120 °C, there was a small decline in solid recovery, and the removal of xylan increased gradually to 30.0%. However, obvious changes in glucan recovery and delignification were not observed. When the pretreatment temperature was ascended to 160 °C, the recoveries of solid and glucan were decreased gradually to 84.8% and 91.6%, respectively. At the same time, the removal of xylan and AIL increased to 37.4% and 16.3%. This phenomenon indicated that delignification was not sensitive to the temperature during 3% NaOH pretreatment.

**Table 1:** Chemical composition of sugarcane bagasse before and after pretreatment under different conditions

Pretreatment conditions	Solid recovery	Glucan (%)		Xylan (%)		AIL (%)	
		Content	Recovery	Content	Removal	Content	Removal
Raw material	–	41.2	–	20.2	–	22.0	–
60 °C + NaOH	90.4	44.9	98.5	16.6	25.5	21.4	11.9
60 °C + NaOH + 6.25% H_2_O_2_	86.3	44.3	92.8	14.9	36.2	21.0	17.6
120 °C + NaOH	89.9	45.2	98.6	15.7	30.0	21.8	11.0
120 °C + NaOH + 6.25% H_2_O_2_	76.5	48.6	90.3	13.6	48.7	21.3	26.0
160 °C + NaOH	84.8	44.7	91.6	14.9	37.4	21.7	16.3
160 °C + NaOH + 6.25% H_2_O_2_	59.6	60.1	87.1	5.2	84.7	22.6	38.9

When AHP pretreatment was conducted at 60 °C for 4 h with 3% NaOH and 6.25% H_2_O_2_, the solid recovery was 86.3%, and the removal of xylan and AIL reached 36.2% and 17.6%, which was higher than that pretreated with only NaOH. This phenomenon was ascribed to the hydroxyl radicals produced from the decomposition of H_2_O_2_ at alkali condition which promoted the deconstruction of hemicellulose and lignin [16]. When the pretreatment temperature was elevated to 120 °C, the solid recovery decreased to 76.5%, ascribing to the widespread degradation of xylan (48.7%) and AIL (26%). However, 48.6% of glucan could be detected in pretreated substrate, indicating that 90% of glucan were retained after pretreatment. As the pretreatment temperature went on increasing to 160 °C, the solid recovery decreased sharply to 59.6% because of the large removal of xylan (84.7%), suggesting that hemicellulose were effectively extracted during AHP pretreatment [17]. Though the glucan content in pretreated solid increased to 60.1%, the recovery decreased to 87.1%. Meanwhile, a decrease of AIL recovery was observed, which might be ascribed to the oxidation of phenolic and carbonyl structures in lignin by hydroxyl or superoxide anion radicals produced from the decomposition of H_2_O_2_ [18]. From these outcomes, it could know that the decomposition of hemicellulose and lignin was promoted with higher reaction temperature, especially in AHP pretreatment. The large degradation of hemicellulose/lignin resulted in the increment of surface area and pore volume, which allowed for the cellulase penetration and the enhancement of enzymatic saccharification [17, 19].

### Characterization of untreated and pretreated sugarcane bagasse

**Fig. 1:**
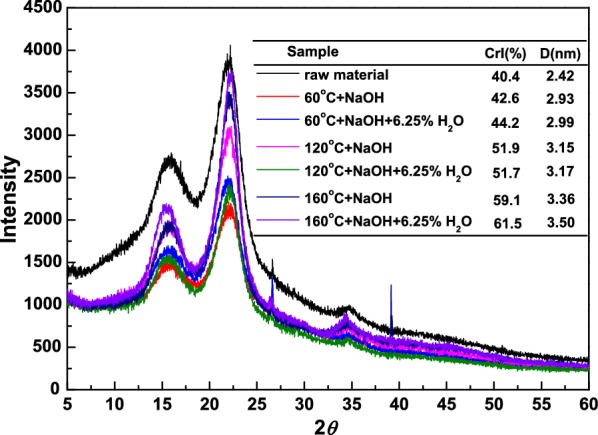
The crystallinity indexes of raw sugarcane bagasse and pretreated substrates

X-ray diffraction curves of untreated and pretreated substrates were used to calculate the crystallinity and cellulose crystallites size, and the results were depicted in Fig. 1. The CrI of native material was 40.4%. After the 3% NaOH pretreatment at 60 °C, the CrI increased to 42.6%. This phenomenon was due to the dissolution/degradation of amorphous hemicellulose and lignin, leading to the increased content of cellulose in pretreated substrates, as confirmed in Table 1 [20]. For NaOH pretreatment, as the temperature increased from 120 to 160 °C, the CrI increased gradually from 51.9 to 59.1%. However, no distinct change in cellulose content was observed. This could be ascribed that more amorphous cellulose were removed at higher pretreatment temperature, and the intensity of crystalline peaks at 15.5° and 22.0°, corresponding to the crystalline cellulose, increased gradually with the elevation of pretreatment temperature. For AHP pretreatment at 60 °C, the CrI increased to 44.2%, which was a little higher than that with only NaOH, attributing to the larger removal of xylan, AIL, and amorphous cellulose. As the pretreatment temperature was increased from 120 to 160 °C, the CrI increased from 51.7 to 61.5%, attributing to the enhancement of cellulose content and the removal of amorphous cellulose. Due to the degradation of hemicellulose, lignin, and amorphous cellulose, the intact structure composed of three main constitutes was ruptured, providing more cellulose fibers for the accessibility of enzyme, and enhancing the enzymatic digestibility [21].

In addition, the average cellulose crystallite size (D) was determined according to the Scherrer equation. As shown in Fig. 1, the average size of untreated material was 2.42 nm (002). After the NaOH pretreatment at 60 °C, its average size reached 2.93 nm, which was larger than that of native material. As the pretreatment temperature was raised from 120 to 160 °C, the average size went on increasing gradually to 3.36 nm. This result was not agreed with that the crystalline size became smaller after pretreatment due to the disruption of cellulose crystallinity [22, 23]. Hence, it was presumed that the crystalline cellulose might be reformed or recrystallized after NaOH pretreatment, leading to the increment of average size of cellulose crystallite [24, 25]. For AHP pretreatment, as the pretreatment temperature was elevated, the average size presented the same tendency with NaOH-pretreated substrates, increased gradually from 2.99 to 3.50 nm.

To investigate the changes of morphological structure after pretreatment, SEM images of untreated and pretreated substrates were observed and presented in Additional file 1: Figure S1. The raw sugarcane bagasse showed an intact and smooth surface with well-ordered fiber bundles, which was not easy for the accessibility of enzyme to cellulose. After NaOH pretreatment or AHP pretreatment at 60 °C, the fibers became rough, loose, and disrupt with cracks, pores, and debris on the surface [7]. These changes could be ascribed to the degradation of hemicellulose/lignin due to the fibers swelling, linkages disruption between lignin and hemicellulose, as well as the radical reaction of OH and lignin [18, 26]. As pretreatment temperature raised from 60 to 160 °C, most of the fibers were disconnected and well-separated with more obvious pores and fragments appeared on the surface of substrates, which could reduce the structure barrier and provide more surface for the accessibility of cellulase, and improve the subsequent enzymatic saccharification [23].

FT-IR spectroscopy (Additional file 1: Figure S2) was used to analysis the structural and chemical changes of sugarcane bagasse after pretreatment. The prominent peaks at 1740 cm^−1^ attributed to the vibrations of acetyl/carbonyl groups in hemicellulose or carboxylic groups in lignin. It became weaken after NaOH or AHP pretreatment, and almost disappeared after pretreated at 160 °C, suggesting that the carboxylic and ester bonds in hemicellulose and lignin were ruptured [7, 27]. The signals of the aromatic lignin ring at 1510 cm^−1^ and 1604 cm^−1^ became intensive after pretreatment. This result suggested that the hemicellulose degradation was faster than delignification during NaOH or AHP pretreatment, which did not decrease the lignin content in pretreated substrates. The peak at 898 cm^−1^ attributed to the β-glycosidic linkages in cellulose, became intensive after pretreatment, which was in agreement with the increment of glucan after pretreatment, as confirmed in Table 1 [28]. Taken together, the FT-IR analysis was in accordance with the chemical analysis data, retained the major cellulose in pretreated substrates by selective breaking functional groups and chemical bonds, and presented an enhanced exposure of cellulose for enzyme accessibility because of the degradation of hemicellulose and lignin [7].

**Fig. 2:**
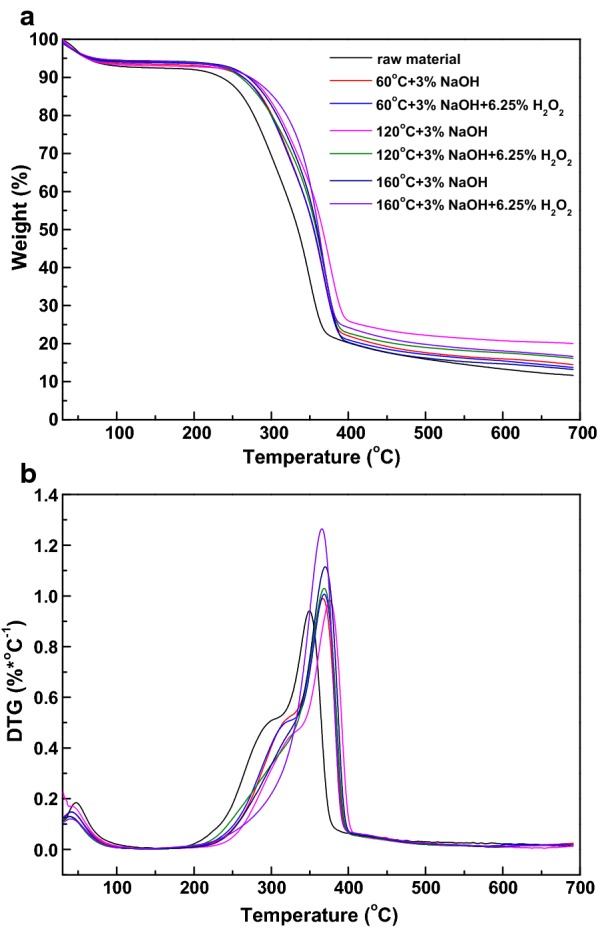
TG and DTG distributions of raw material and pretreated substrates

As is known, the thermal properties of lignocellulosic biomass associated with the chemical constitute and their structural characteristics [29]. TG and DTG analyses of untreated and pretreated substrates were detected and illustrated in Fig. 2. As shown, the original weight loss between 5.5 and 7.2% below 120 °C was corresponded to the evaporation of moisture. All samples adequately degraded between 200 and 400 °C, including hemicellulose decomposition (180–320 °C), cellulose decomposition (320–400 °C), and lignin decomposition (> 360 °C) [7]. It was noteworthy that pretreated substrates presented more weight loss than the native material, which was in line with the chemical constitutes shown in Table 1 that pretreated samples contained more cellulose and lignin but less hemicellulose compared with untreated material.

For native material, two main weight loss peaks could be observed during DTG analysis. The first one was at 302 °C with DTG of 0.51% C^−1^, ascribing to hemicellulose degradation. The second one occurred at 350 °C with DTG of 0.95% C^−1^ due to the decomposition of cellulose/lignin, indicating that the decomposition of cellulose/lignin were more refractory than that of hemicellulose [30]. After NaOH or AHP pretreatment at 60 °C or 120 °C, the DTG presented similar curves (two main weight loss peaks) with raw material. However, as the pretreatment temperature increased from 60 to 120 °C, the first peak became diminished gradually because of the removal of hemicellulose. This phenomenon indicated that NaOH/AHP pretreatment modified the biomass compositions, leading to the appearance of specific properties revealed by the DTG curves. However, when the pretreatment temperature was elevated to 160 °C, there was only one main weight loss peak on DTG curves, which was consistent with the degradation of cellulose/lignin. The maximum temperatures of weight loss peaks for the NaOH- and AHP-pretreated substrates at 60, 120, and 160 °C were 367, 369, 376, 369, 370, and 366 °C, respectively, which were all higher than that of raw material (350 °C). This phenomenon could be concluded that the degradation of partial hemicellulose and lignin during NaOH or AHP pretreatment increased the proportion of crystalline cellulose, as shown in Fig. 1. Cellulose crystals are thermally stable due to its rich hydrogen bonds, strong cellulose chains, and high polymerization degree, which contributed to the higher decomposition temperature and thermal stability of pretreated samples [7]. Furthermore, the residual lignin in pretreated substrates might also increase the decomposition temperature.

### Effect of pretreatment conditions on enzymatic saccharification

**Fig. 3:**
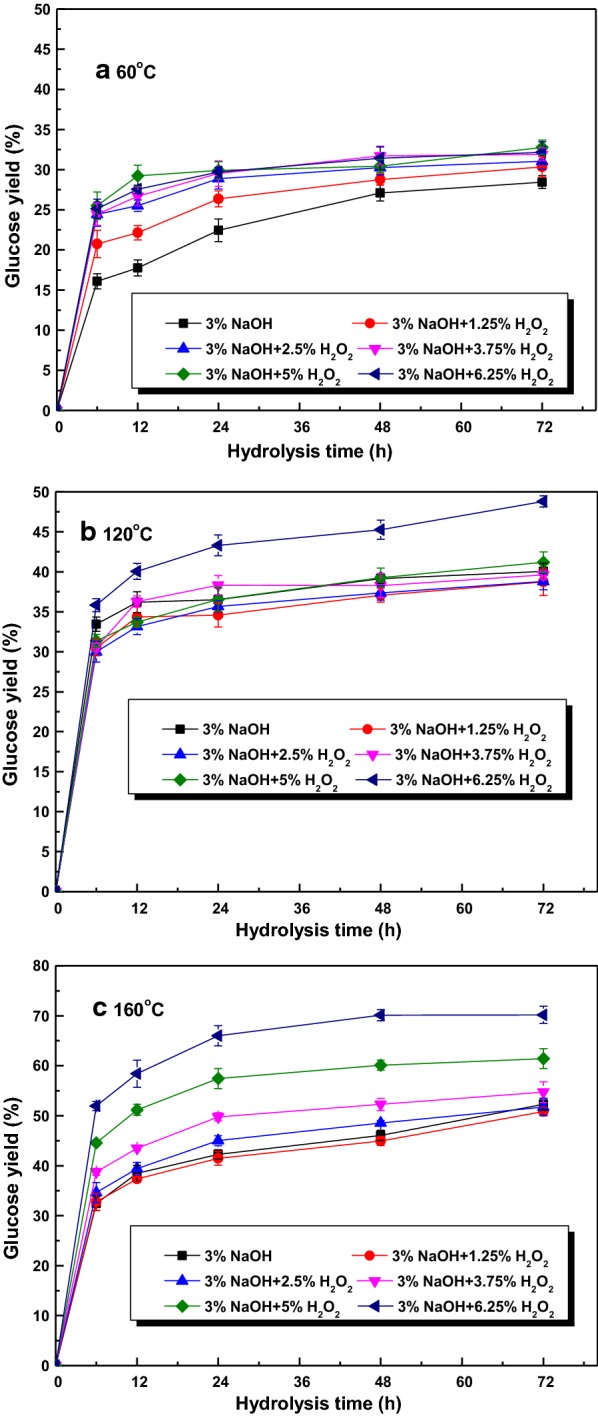
Glucose yield after NaOH and AHP pretreatment under different temperatures

Based on the above investigation, it is revealed that the crystallinity, average size, surface area, redistribution of components contents in sugarcane bagasse were greatly modified by the NaOH and AHP pretreatment. Hence, to explore the influence of structural and compositional changes on enzymatic saccharification, the glucose yields of pretreated substrates as a function of incubation time were investigated and presented in Fig. 3. For native material, the glucose yield went up gradually to 22.4% after 72 h [13]. After pretreatment, all samples performed higher glucose yield than the native material. However, when the pretreatment temperature was 60 °C, only a marginal improvement in glucose yield could be observed no matter for NaOH or AHP-pretreated substrates. Increasing the H_2_O_2_ loading during AHP pretreatment did not lead to great enhancement in enzymatic hydrolysis. This phenomenon was ascribed to the slight degradation of xylan and lignin, which did not cause extensive breakdown of the intact structure of native material [18]. When the pretreatment temperature was risen to 120 °C, the NaOH-pretreated solid yielded glucose yield about 40% of after 72 h. When AHP pretreatment were conducted with 1.25–3.75% H_2_O_2_, the glucose yields were a little lower than that obtained from NaOH substrate [10]. As the H_2_O_2_ loading increased to 6.25%, the glucose yield increased gradually to 48.8%, attributing to the degradation of hemicellulose (48.7%) and AIL (26%) over pretreatment process, which provided more cellulose for the accessibility of enzyme and was in consistent with the previous literatures [18, 26]. When pretreatment temperature of 160 °C was implemented, 52.3% of glucose could be yielded after 72 h for NaOH-pretreated substrate. For AHP pretreatment, the glucose yield showed an increased tendency with the increment of H_2_O_2_ dosage, and reached the highest yield of 70.2% with 6.25% H_2_O_2_, representing 80.7% of glucose in pretreated substrate. This enhancement was due to the potential effect of AHP pretreatment on delignification (38.9%) and hemicellulose removal (84.7%) without significant cellulose degradation (< 13%), which damaged the intact structure, increased porosity and surface area, made it easy for the accessibility of cellulase [31–33].

**Fig. 4:**
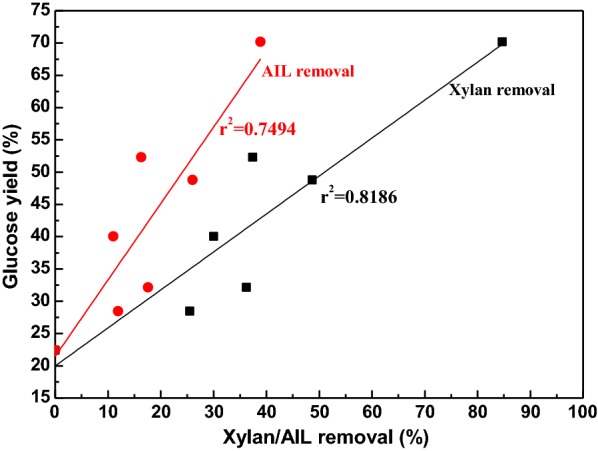
Correlation between glucose yield and xylan/AIL removal

According to the results in Fig. 3, enzymatic saccharification of pretreated substrate was accelerated greatly because of the degradation of hemicellulose and lignin, leading to the increment of porosity of biomass, providing more cellulose for the accessibility of cellulase, reducing the nonspecific bonding to enzyme, thus enhancing the enzymatic digestibility. To determine that the removal of hemicellulose/lignin were positively related to the enhancement of enzymatic saccharification, the glucose yields were plotted against the removal of xylan/AIL, and the results were depicted in Fig. 4. When there was no lignin and hemicellulose removal, that is to say, native material was regarded as substrate for enzymatic saccharification, only 22.4% of glucose could be obtained. A clear relationship was consistently observed between the removal of xylan/AIL and glucose yield. When the removal of xylan/AIL were increased from 25.5 to 84.7% and 11.9 to 38.9%, respectively, the glucose yields linearly and positively increased from 28.4 to 70.2% with *r*^2^ of 0.7494 and 0.8186, respectively. Similar phenomenon was observed for the H_2_O_2_–Na_2_CO_3_ pretreatment of corn stover [34].

### The enhanced production of glucose with Tween 80

**Fig. 5:**
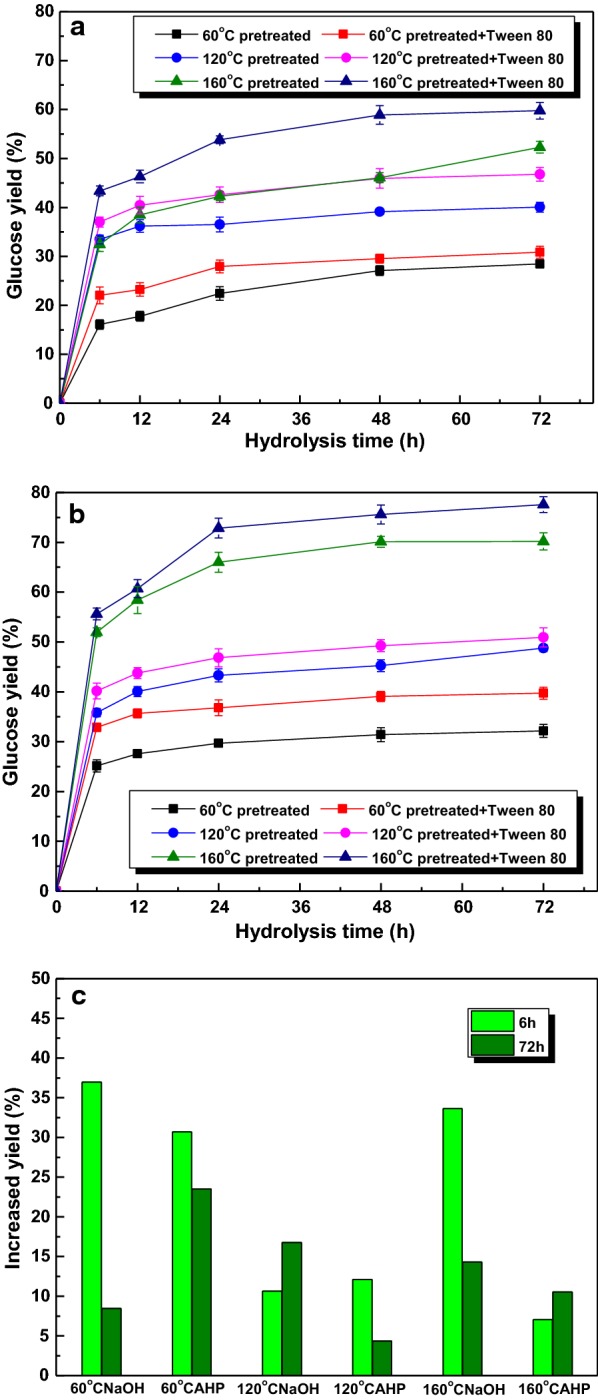
Glucose yields and increased yields obtained from different pretreated substrates with the addition of Tween 80 (**a** NaOH-pretreated substrates, **b** NaOH + 6.25% H_2_O_2_ pretreated substrates, **c** increased yields with Tween 80)

In order to go on improving the enzymatic efficiency, several subsequent experiments with Tween 80 (150 mg/g substrate) were conducted, and the results were illustrated in Fig. 5. The glucose yields of all samples were enhanced greatly with Tween 80. For substrates pretreated with NaOH or AHP at 60 °C, the control glucose yields after 6 h without additive were 16.1% and 25.1%, respectively. With the addition of Tween 80, the glucose yields increased to 22.0% and 32.8% with the increased yields of 36.6% and 30.7%, respectively. This enhancement could be concluded that the addition of Tween 80 reduced the cellulase adsorption on lignin by hydrophobic interactions, increased desorption of cellulase in the substrate fibers, provided more cellulase for enzymatic saccharification, resulting in the improvement of enzymatic efficiency [33, 35, 36]. After hydrolysis for 72 h, the glucose yields with Tween 80 increased gradually to 30.8% and 39.7%, respectively, which were higher than that without Tween 80 (28.4% and 32.2%) [37]. However, the increased yields did not increase, but reduced to 8.4% and 23.5%, respectively. This phenomenon could be interpreted in two ways. First, there was not enough cellulase provided for cellulose hydrolysis because of the non-desorption of it from enzymatic substrate [21]. Second, substrates became more recalcitrant. The residual cellulose was enriched in crystalline portions encapsulated by lignin and hemicellulose, the mild pretreatment conditions were not enough for the dissolution of it, which impeded the enzymatic efficiency [13, 33].

When the pretreatment temperature was increased to 120 °C, for NaOH-pretreated solid, the glucose yields increased gradually with extended time. However, the increased yields with Tween 80 presented the contrary tendency with other substrates, increased gradually from 10.6 to 16.8% over time from 6 h to 72 h. When AHP-pretreated substrate was conducted, the glucose yields with Tween 80 increased gradually to 50.9% after 72 h, and the increased yields decreased from 12.1% (6 h) to 4.4% (72 h), which presented the same tendency with that pretreated at 60 °C using AHP pretreatment. For substrate pretreated at 160 °C with NaOH, with the addition Tween 80, the glucose yields after 72 h increased from 52.3 to 59.8%. And the increased yields decreased greatly from 33.6% to 14.3% as the hydrolysis time was prolonged from 6 h to 72 h. A possible reason for this decrease is that the available glucose in pretreated substrate which can be released by enzymatic hydrolysis became less with extended time. When 160 °C AHP-pretreated substrate was used for enzymatic hydrolysis, the glucose yield with Tween 80 after 72 h increased from 70.2 to 77.6%, the highest glucose yield represented 89.1% of glucose in pretreated substrate. However, as the hydrolysis time was stretched, the increased yields of glucose did not decrease, but increased from 7.0 to 10.5%.

Pretreatment was the essential step for the bioconversion of lignocellulosic biomass to bioethanol and bio-chemicals. The results in this study suggested that 3% NaOH and 6.25% H_2_O_2_ pretreatment provided a promising technology to achieve high glucose yield with addition of Tween 80 from sugarcane bagasse. This proposed pretreatment not only generated high glucose yield, but also reduced the cost of the bio-refinery process due to the lower loading of chemicals and gradual conditions.

## Methods

### Materials

Sugarcane bagasse was collected from Shaoguan, China. They were ground using a mill to obtain the powders with diameter below 1 mm. The chemical analysis of native sugarcane bagasse was detected based on the protocol NREL/TP-510-42618 proposed by the NREL [38]. It contained 41.2% cellulose, 22.8% hemicellulose [including 20.2% xylan, 1.8% arabinan, 0.8% galactan), 25.2% lignin (containing 22.0% acid insoluble lignin (AIL), 3.2% acid soluble lignin (ASL)], and 3.6% ash.

### Pretreatment and enzymatic hydrolysis

The operating conditions of AHP pretreatment were as follows: 3 wt% NaOH and 1.25–6.25 wt% H_2_O_2_ (both based on dry sugarcane bagasse), temperature 60–160 °C, time 1–4 h, and solid/liquid ratio of 1:10. Meanwhile, the pretreatment with only NaOH (3 wt%) under different temperatures were also carried out. The mild pretreatment conditions of 60 °C for 4 h were employed in a shaker. However, the higher pretreatment reactions at 120 °C for 2 h or 160 °C for 1 h were tested in a 1 L Parr reactor. After the reaction was completed, iced water was used to stop the reaction immediately. Then, the solids were separated by filtration and washed thoroughly with de-ionized water, and stored in refrigerator for further use.

The enzymatic saccharification experiments were performed at 2% (w/v) solid loading in 50 mM sodium acetate buffer (pH 4.8) in a 250 mL Erlenmeyer flask. Cellic CTec2, with a cellulytic activity of 90 FPU/mL, was added at loading of 20 FPU/g dry substrate. Then, the mixtures were incubated in a shaker at 50 °C and 150 rpm [13]. After enzymatic saccharification for 6, 12, 24, 48, and 72 h, a small amount of liquor was taken out from the hybrid and centrifuged at 10,000 rpm for several minutes to quantify the sugars. To determine the enhancement of additive on enzymatic saccharification, 150 mg Tween 80 was acceded to the hybrid based on per g dry substrate. For the complete interaction between Tween 80 and substrate, they should be incubated with hybrid for 30 min before the addition of cellulase.

### Analytical methods

The chemical compositions of untreated and pretreated solids, and sugars obtained from enzymatic saccharification were determined by HPLC (Shimadzu, Japan) equipped with a of SUGAR SH1011 column and a refractive index detector at 50 °C with 1.0 mL/min of 5 mM H_2_SO_4_ as eluates [39]. The glucose yield obtained from enzymatic hydrolysis and the increased yields of glucose with Tween 80 were determined based on the following equations:

$$ {\text{Glucose}}\;{\text{yield }}(\% ) = \frac{{{\text{Glucose}}\;{\text{produced}}\;{\text{in}}\;{\text{enzymatic}}\;{\text{hydrolysis}}}}{{{\text{Glucan}}\;{\text{amount}}\;{\text{in}}\;{\text{raw}}\;{\text{material}}*1.11}} \times 100\% $$

$$ {\text{Increased}}\;{\text{yield }}(\% ) = \frac{{{\text{Glucose}}\;{\text{yield}}\;{\text{with}}\;{\text{surfactant}} - {\text{glucose}}\;{\text{yield}}\;{\text{without}}\;{\text{surfactant}}}}{{{\text{Glucose}}\;{\text{yield}}\;{\text{without}}\;{\text{surfactant}}}} \times 100\% . $$

### Characterization of untreated and pretreated solids

Cellulose crystallinity and the average crystallite size (D) of untreated and pretreated sugarcane bagasse were detected by an X-ray diffractometer (D8-ADVANCE, Bruker, Germany) using Cu radiation (*k* = 0.1541 nm). The scattering angles (2*θ*) ranged from 5° to 60°. The CrI and crystallite size (D) were calculated based on Segal method and Scherrer equation, respectively [13, 32, 40]. SEM was used to measure the morphological changes of substrates (FEI Verios 460, USA). The FT-IR spectroscopy was evaluated by a Tensor 27 FT-IR spectrometer (Bruker, Germany) in the range of resolution between 4000 and 400 cm^−1^. The thermogravimetric and differential thermogravimetric (TG/DTG) analysis was conducted with an analyzer TG-Q500 (TA instruments, USA) under N_2_ atmosphere.

## Conclusions

AHP pretreatment of sugarcane bagasse under different temperatures was applied to enhance enzymatic saccharification. It was demonstrated that there was a positive correlation between H_2_O_2_ loading and glucose yield, ascribing to the widespread degradation of hemicellulose and lignin, which disrupted the matrix structure and provided more cellulose for the accessibility of cellulase. Moreover, the addition of Tween 80 played remarkable performances in promoting the glucose yield during enzymatic hydrolysis by stabilizing and protecting the enzyme activity. The highest glucose yield of 77.6% was obtained after pretreatment at 160 °C for 60 min with 6.25% H_2_O_2_ and the addition of Tween 80, representing 89.1% of glucose in pretreated substrate.

## Additional file

**Additional file 1: Figure S1.** SEM images of raw material and pretreated substrates at ×2000 magnification. **Figure S2.** FTIR spectra of untreated and pretreated samples with different pretreatment conditions.

## References

[1] Mokomele T, da Sousa Costa L, Balan V, van Rensburg E, Dale BE, Görgens JF (2018). Ethanol production potential from AFEX™ and steam-exploded sugarcane residues for sugarcane biorefineries. Biotechnol Biofuels.

[2] Batalha LAR, Han Q, Jameel H, Chang HM, Colodette JL, Gomes FJB (2015). Production of fermentable sugars from sugarcane bagasse by enzymatic hydrolysis after autohydrolysis and mechanical refining. Bioresour Technol.

[3] Ji Z, Zhang X, Ling Z, Zhou X, Ramaswamy S, Xu F (2015). Visualization of *Miscanthus* x *giganteus* cell wall deconstruction subjected to dilute acid pretreatment for enhanced enzymatic digestibility. Biotechnol Biofuels.

[4] Auxenfans T, Cronier D, Chabbert B, Paes G (2017). Understanding the structural and chemical changes of plant biomass following steam explosion pretreatment. Biotechnol Biofuels.

[5] Alvira P, Tomas-Pejo E, Ballesteros M, Negro MJ (2010). Pretreatment technologies for an efficient bioethanol production process based on enzymatic hydrolysis: a review. Bioresour Technol.

[6] Li M, Pattathil S, Hahncde MG, Hodge DB (2014). Identification of features associated with plant cell wall recalcitrance to pretreatment by alkaline hydrogen peroxide in diverse bioenergy feedstocks using glycome profiling. RSC Adv.

[7] Phitsuwan P, Sakka K, Ratanakhanokchai K (2016). Structural changes and enzymatic response of Napier grass (*Pennisetum purpureum*) stem induced by alkaline pretreatment. Bioresour Technol.

[8] Bansal N, Bhalla A, Pattathil S, Adelman SL, Hahn MG, Hodge DB, Hegg EL (2016). Cell wall-associated transition metals improve alkaline-oxidative pretreatment in diverse hardwoods. Green Chem.

[9] Li M, Wang J, Yang Y, Xie G (2016). Alkali-based pretreatments distinctively extract lignin and pectin for enhancing biomass saccharification by altering cellulose features in sugar-rich Jerusalem artichoke stem. Bioresour Technol.

[10] Alvarez-Vasco C, Zhang X (2017). Alkaline hydrogen peroxide (AHP) pretreatment of softwood: enhanced enzymatic hydrolysability at low peroxide loadings. Biomass Bioenergy.

[11] Mittal A, Katahira R, Donohoe BS, Black BA, Pattathil S, Stringer JM, Beckham GT (2017). Alkaline peroxide delignification of corn stover. ACS Sustain Chem Eng.

[12] Yuan ZY, Wen YB, Kapu NS (2018). Ethanol production from bamboo using mild alkaline pre-extraction followed by alkaline hydrogen peroxide pretreatment. Bioresour Technol.

[13] Zhang H, Fan M, Li X, Zhang A, Xie J (2018). Enhancing enzymatic hydrolysis of sugarcane bagasse by ferric chloride catalyzed organosolv pretreatment and Tween 80. Bioresour Technol.

[14] Yan L, Zhang L, Yang B (2014). Enhancement of total sugar and lignin yields through dissolution of poplar wood by hot water and dilute acid flow through pretreatment. Biotechnol Biofuels.

[15] Kim W, Gamo Y, Sani YM, Wusiman Y, Ogawa S, Karita S, Goto M (2006). Effect of Tween 80 on hydrolytic activity and substrate accessibility of carbohydrolase I (CBH I) from *Trichoderma viride*. Asian Australas J Anim.

[16] Rabetafika HN, Bchir B, Blecker C, Paquot M, Wathelet B (2014). Comparative study of alkaline extraction process of hemicelluloses from pear pomace. Biomass Bioenergy.

[17] Song X, Jiang Y, Rong X, Wei W, Wang S, Nie S (2016). Surface characterization and chemical analysis of bamboo substrates pretreated by alkali hydrogen peroxide. Bioresour Technol.

[18] Cabrera E, Munoz MJ, Martin R, Caro I, Curbelo C, Diaz AB (2014). Alkaline and alkaline peroxide pretreatments at mild temperature to enhance enzymatic hydrolysis of rice hulls and straw. Bioresour Technol.

[19] Alvarez-Vasco C, Zhang X (2013). Alkaline hydrogen peroxide pretreatment of softwood: hemicellulose degradation pathways. Bioresour Technol.

[20] Liu H, Pang B, Zhao YD, Lu J, Han Y, Wang HS (2018). Comparative study of two different alkali-mechanical pretreatments of corn stover for bioethanol production. Fuel.

[21] Kumar R, Wyman CE (2009). Cellulase adsorption and relationship to features for corn stover solids produced by leading pretreatments. Biotechnol Bioeng.

[22] Zhang JZ, Ma XX, Yu JL, Zhang X, Tan TW (2011). The effects of four different pretreatments on enzymatic hydrolysis of sweet sorghum bagasse. Bioresour Technol.

[23] Sun FB, Wang L, Hong JP, Ren JL, Du FG, Hu JG, Zhang ZY, Zhou BW (2015). The impact of glycerol organosolv pretreatment on the chemistry and enzymatic hydrolyzability of wheat straw. Bioresour Technol.

[24] Tang S, Liu R, Sun FF, Dong C, Wang R, Gao Z, Zhang Z, Xiao Z, Li C, Li H (2017). Bioprocessing of tea oil fruit hull with acetic acid organosolv pretreatment in combination with alkaline H_2_O_2_. Biotechnol Biofuels.

[25] Gurgel LVA, Marabezi K, Ramos LA, Curvelo AADS (2012). Characterization of depolymerized residues from extremely low acid hydrolysis (ELA) of sugarcane bagasse cellulose: effects of degree of polymerization, crystallinity and crystallite size on thermal decomposition. Ind Crop Prod.

[26] Correia JAD, Marques JE, Goncalves LRB, Rocha MVP (2013). Alkaline hydrogen peroxide pretreatment of cashew apple bagasse for ethanol production: study of parameters. Bioresour Technol.

[27] Flauzino Neto WP, Silverio HA, Dantas NO, Pasquini D (2013). Extraction and characterization of cellulose nanocrystals from agro-industrial residue-soy hulls. Ind Crop Prod.

[28] Ravindran R, Sarangapani C, Jaiswal S, Cullen PJ, Jaiswal AK (2017). Ferric chloride assisted plasma pretreatment of lignocellulose. Bioresour Technol.

[29] Grimaldi MP, Marques MP, Laluce C, Cilli EM, Sponchiado SRP (2015). Evaluation of lime and hydrothermal pretreatments for efficient enzymatic hydrolysis of raw sugarcane bagasse. Biotechnol Biofuels.

[30] Yu J, Paterson N, Blamey J, Millan M (2017). Cellulose, xylan and lignin interactions during pyrolysis of lignocellulosic biomass. Fuel.

[31] Yuan TQ, Wang W, Zhang LM, Sun RC (2013). Reconstitution of cellulose and lignin after [C2mim][OAc] pretreatment and its relation to enzymatic hydrolysis. Biotechnol Bioeng.

[32] Zhang H, Wu S (2015). Generation of lignin and enzymatically digestible cellulose from ethanol-based organosolv pretreatment of sugarcane bagasse. Cellulose.

[33] Mesquita JF, Ferraz A, Aguiar A (2016). Alkaline-sulfite pretreatment and use of surfactants during enzymatic hydrolysis to enhance ethanol production from sugarcane bagasse. Bioprocess Biosyst Eng.

[34] Gong W, Liu C, Mu X, Du H, Lv D, Li B, Han S (2015). Hydrogen peroxide-assisted sodium carbonate pretreatment for the enhancement of enzymatic saccharification of corn stover. ACS Sustain Chem Eng.

[35] Zhao X, Zhang L, Liu D (2012). Biomass recalcitrance Part I: the chemical compositions and physical structures affecting the enzymatic hydrolysis of lignocellulose. Biofuels Bioprod Biorefin.

[36] Li Y, Sun Z, Ge X, Zhang J (2016). Effects of lignin and surfactant on adsorption and hydrolysis of cellulases on cellulose. Biotechnol Biofuels.

[37] Jin W, Chen L, Hu M, Sun D, Li A, Li Y, Hu Z, Zhou S, Tu Y, Xia T, Wang Y, Xie G, Li Y, Bai B, Peng L (2016). Tween-80 is effective for enhancing steamexploded biomass enzymatic saccharification and ethanol production by specifically lessening cellulase absorption with lignin in common reed. Appl Energy.

[38] Sluiter A, Hames B, Ruiz R, Scarlata C, Sluiter J, Templeton D, Crocker D. Determination of structural carbohydrates and lignin in biomass. National Renewable Energy Laboratory; 2008.

[39] Yu Q, Tan X, Zhuang X, Wang Q, Wang W, Qi W, Zhou G, Luo Y, Yuan Z (2016). Co-extraction of soluble and insoluble sugars from energy sorghum based on a hydrothermal hydrolysis process. Bioresour Technol.

[40] Segal L, Creely L, Martin AE (1959). An empirical method for estimating the degree of crystallinity of native cellulose using X-ray diffractometer. Text Res J.

