# Supplementary material for: Investigation of alkaline hydrogen peroxide pretreatment and Tween 80 to enhance enzymatic hydrolysis of sugarcane bagasse
Source: Biotechnol Biofuels. 2019 May 3;12:107. doi: 10.1186/s13068-019-1454-3 (PMC6498686; doi:10.1186/s13068-019-1454-3)
Supplement: Supplementary file 1 — Additional file 1: Figure S1. SEM images of raw material and pretreated substrates at ×2000 magnification. Figure S2. FTIR spectra of untreated and pretreated samples with different pretreatment conditions. [file 13068_2019_1454_MOESM1_ESM.docx]

**Investigation of** **alkaline hydrogen peroxide pretreatment and Tween 80 to enhance enzymatic hydrolysis of sugarcane bagasse**

**Hongdan Zhang^a,b*^, Shihang Huang^a^, Weiqi Wei^c^, Jiajie Zhang^a^, Jun Xie^a^**

^a^ College of Forestry and Landscape Architecture, Key Laboratory of Energy Plants Resource and Utilization, Ministry of Agriculture, South China Agricultural University, Guangzhou 510642, P.R. China

^b^ CAS Key Laboratory of Renewable Energy, Guangzhou Institute of Energy Conversion, Guangzhou 510640, P.R. China

c College of Light Industry and Food Engineering, Nanjing Forestry University, Nanjing 210037, P.R. China

Corresponding author: Hongdan Zhang; E-mail address: [hdzhang@scau.edu.cn](mailto:hdzhang@scau.edu.cn).


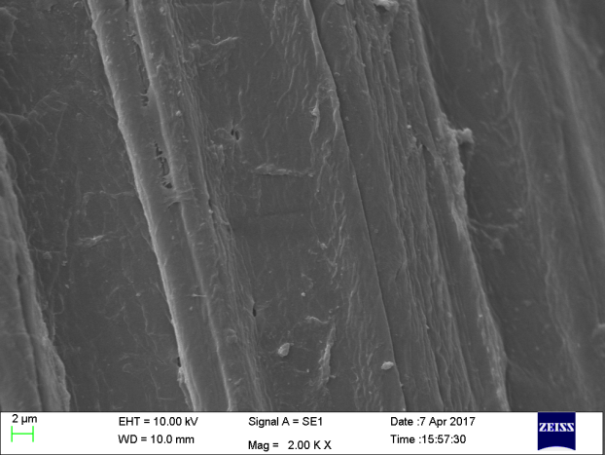
**
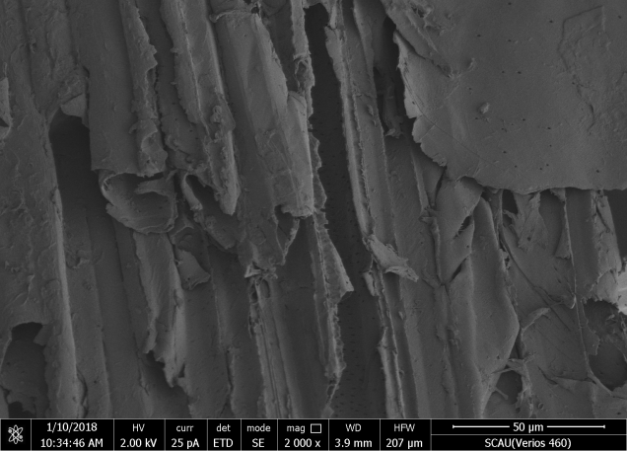
**

**Raw material**

**60^o^C+NaOH**

**
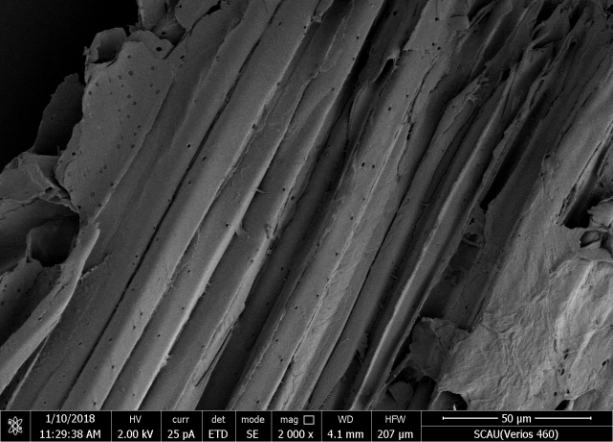

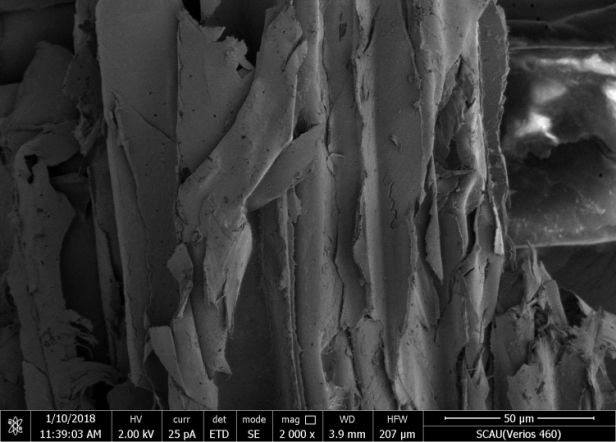
**

**120^o^C+NaOH**

**60^o^C+NaOH+6.25%H_2_O_2_**

**
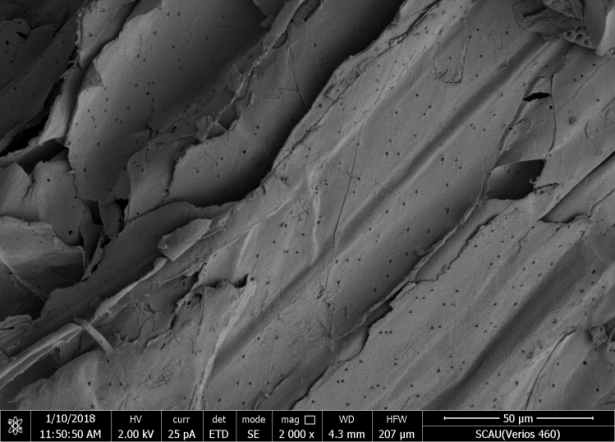

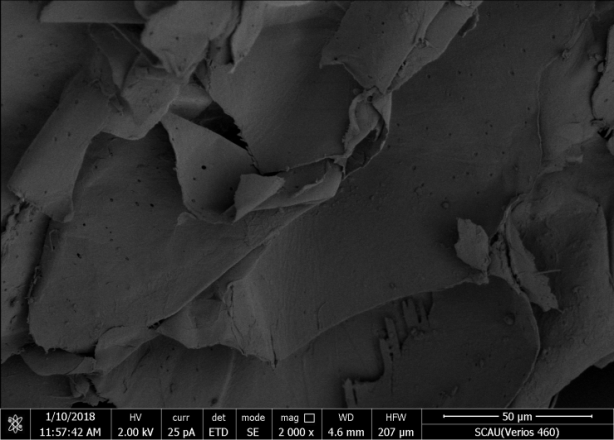
**

**120^o^C+NaOH+6.25%H_2_O_2_**

**160^o^C+NaOH**

**
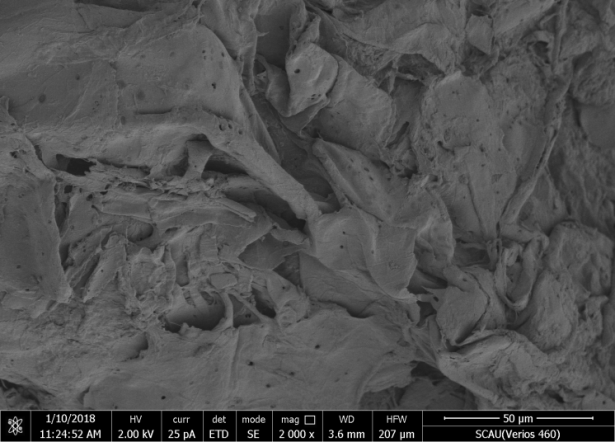
**

**160^o^C+NaOH+6.25%H_2_O_2_**

**Figure S1.** SEM images of raw material and pretreated substrates at ×2000 magnification.

**Figure S2.** FTIR spectra of untreated and pretreated samples with different pretreatment conditions.
